# A rare case of congenital appendiceal duplication complicated by an appendiceal-sigmoid fistula: A case report

**DOI:** 10.1016/j.ijscr.2025.111429

**Published:** 2025-05-13

**Authors:** Suleiman Ayalew Belay, Michael A. Negussie, Melaku Tessema Kassie, Yishak Abdulsemed, Filimon Getaneh Assefa, Fuad Seid Ebrahim

**Affiliations:** a School of Medicine, College of Medicine and Health Sciences, University of Gondar, Gondar, Ethiopia; b School of Medicine, College of Health Sciences, Addis Ababa University, Addis Ababa, Ethiopia; c Department of Surgery, School of Medicine, College of Medicine and Health Sciences, University of Gondar, Gondar, Ethiopia

**Keywords:** Appendiceal duplication, Appendiceal-sigmoid fistula, Congenital anomaly, Case report

## Abstract

**Introduction:**

Duplication of the appendix is a rare congenital anomaly, occurring in approximately 0.004 % to 0.009 % of the population. In contrast, appendiceal-sigmoid fistulas are uncommon acquired conditions, usually associated with chronic inflammation or neoplastic processes. To our knowledge, the coexistence of these two entities has not been previously reported.

**Case presentation:**

A 26-year-old female presented with a one-month history of right lower quadrant abdominal pain, loss of appetite, and low-grade fever. Imaging findings initially suggested chronic appendicitis. During exploratory laparotomy, a Type A appendiceal duplication was discovered, with one of the appendices forming a fistulous connection to the sigmoid colon. Surgical treatment involved appendectomy, excision of the fistula, and primary repair of the colonic defect. Histopathological examination confirmed the diagnosis of duplicated appendix with chronic inflammatory changes. The patient had an uneventful postoperative recovery.

**Discussion:**

Appendiceal duplication typically remains an incidental finding, rarely causing clinical symptoms. The coexistence of appendiceal duplication and sigmoid fistula formation haven't been previously reported. Diagnosis can be challenging due to the limitations of preoperative imaging in identifying these anomalies. Therefore, surgical exploration remains crucial, particularly in symptomatic patients, to manage symptoms and prevent potential complications.

**Conclusion:**

Clinicians should maintain a high index of suspicion for rare congenital anomalies like appendiceal duplication in patients who present with atypical symptoms of appendicitis.

## Highlights

• Appendiceal duplication is a rare anomaly (0.004%–0.009% incidence).
• Appendiceal-sigmoid fistulas are rare and often result from chronic inflammation or tumors.
• First reported case of duplicated appendix with an appendiceal-sigmoid fistula.
• Diagnosis was made intraoperatively due to imaging limitations.
• Surgery led to full recovery after appendectomy and colonic repair.

## 1 Introduction

Appendiceal duplication is an exceptionally rare congenital anomaly, with an incidence ranging from 0.004 % to 0.009 % in appendectomy specimens [1]. In contrast, appendiceal-sigmoid fistulas are typically acquired and occur infrequently, often arising secondary to chronic inflammatory conditions such as appendicitis or appendiceal neoplasms or sigmoid diverticulitis [2,3].

This report presents a unique case of a duplicate appendix associated with an appendiceal-sigmoid fistula, an exceedingly rare combination. To the best of our knowledge, no previous cases of such a concurrent presentation have been documented in the literature.

This case has been reported in accordance with the SCARE criteria [4].

## 2 Case presentation

A 26-year-old female from Gondar, Northern Ethiopia, presented with a one-month history of intermittent abdominal pain, anorexia, and low-grade fever. She had no prior surgical history or significant comorbidities like diabetes mellitus or hypertension. On physical examination, her vital signs were within normal limits. Abdominal examination revealed direct and rebound tenderness localized to the right lower quadrant. However, other signs typically associated with appendicitis, such as the Psoas and Obturator signs, were negative. Laboratory investigations, including a complete blood count (CBC) and C-reactive protein (CRP), were within normal limits. Abdominal ultrasound demonstrated a dilated, non-compressible appendix, suggestive of chronic appendicitis, which prompted the decision to proceed with surgical intervention. Further evaluation with an abdominal CT scan was considered; however, it was temporarily unavailable.

**Fig. 1:**
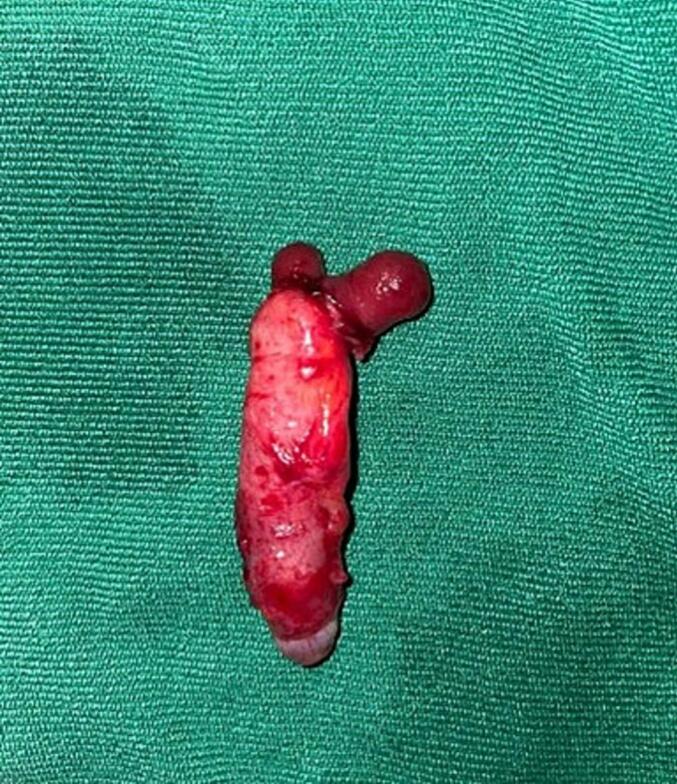
Photograph of the duplicated appendix.

**Fig. 2:**
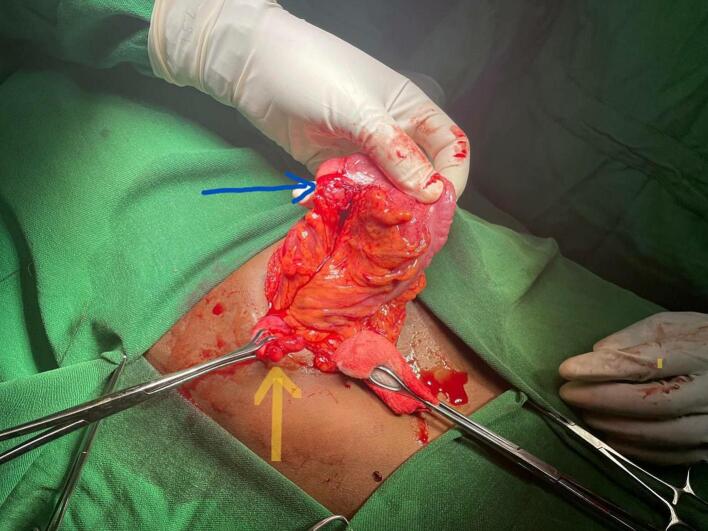
Blue arrow indicating a fistula at the dome of the sigmoid colon; yellow arrow indicating an appendiceal cyst.

Under general anesthesia Right lower quadrant incision was made. Intraoperatively, a duplicate appendix was identified (Fig. 1). One of the appendices was cystic and firmly adhered to the sigmoid colon, forming a fistulous tract between the two structures (Fig. 2). A standard appendectomy was performed, and the fistulous connection was excised. The defect in the sigmoid colon was repaired primarily in two layers using interrupted sutures. Histopathological examination revealed a chronic inflammatory process and confirmed the presence of an appendiceal duplication cyst. Based on the modified Cave-Wallbridge classification, it was categorized as Type A—partial duplication of the appendix arising from a single cecum [5].

The postoperative course was uneventful. The patient remained hemodynamically stable and was discharged on postoperative day five in good condition. She was scheduled for follow-up in the outpatient clinic. At the six-week follow-up, she remained asymptomatic, and a repeat abdominal ultrasound showed no evidence of complications.

## 3 Discussion

The modified Cave-Wallbridge classification categorizes appendiceal duplication into three main types. Type A involves a single cecum with a partially or completely duplicated appendix sharing a common base. Type B includes two completely separate appendices, with B1 (avian type) located symmetrically on either side of the ileocecal valve, and B2 (tenia coli type) where the second appendix arises along the anterior tenia coli. Type C, the rarest form, features two separate ceca, each with its own appendix, sometimes accompanied by additional anomalies like horseshoe or triple appendices [5,10].

In this case, the patient had a Type A duplicated appendix, with one appendix appearing cystic and forming a fistulous tract to the sigmoid colon, while the other was structurally normal. The fistula likely developed as a result of chronic inflammation from longstanding appendicitis. This anatomical variation is thought to arise from incomplete embryological development between the fifth and seventh weeks of gestation, during the formation of the cecum and appendix [5].

A systematic review identified 301 reported cases of appendiceal fistulas [6]. The most frequently involved sites were: bladder (148 cases), skin (40 cases), vasculature (19 cases), umbilicus (16 cases), and gastrointestinal tract (remaining cases). The most common etiology was appendicitis (150 cases), followed by appendiceal adenocarcinoma (32 cases) and congenital abnormalities (18 cases). The condition was more prevalent in males than females, with a 1.7:1 ratio.

A duplicate appendix is often an incidental intraoperative finding, discovered during surgery for other indications or when complications such as appendicitis arise. The clinical presentation of an appendiceal-sigmoid fistula is often nonspecific and may mimic other intra-abdominal pathologies. Patients typically present with abdominal pain, either localized to the right lower quadrant or more generalized. Other associated symptoms include nausea, vomiting, anorexia, and fever, resembling acute appendicitis. When a fistula is present, patients may experience altered bowel habits, such as diarrhea or constipation, due to abnormal communication between the appendix and the sigmoid colon [7,8].

Imaging modalities play a crucial role in the preoperative identification of appendiceal fistulas. Abdominal computed tomography (CT) scans are particularly valuable in detecting fistulas, associated abscesses, or masses contributing to the condition [3,9]. In some cases, colonoscopy can aid in identifying the fistulous opening within the sigmoid colon [8]. However, the diagnosis of a duplicate appendix is typically made intraoperatively, as preoperative imaging may fail to detect the anomaly.

The management of appendiceal-sigmoid fistulas depends on the underlying etiology and associated complications. In asymptomatic or uncomplicated cases, non-operative management may be appropriate, as demonstrated in instances where sigmoid-appendiceal fistulas secondary to diverticulitis were treated conservatively [8]. However, in the presence of infection, malignancy, or obstruction, surgical intervention is warranted. The standard surgical approach involves resection of the appendix, excision of the fistulous tract, and primary repair of the affected segment of the sigmoid colon, similar to the procedure performed in this case.

## 4 Conclusion

Clinicians should maintain a high index of suspicion for rare congenital anomalies such as appendiceal duplication, especially in patients presenting with atypical features of appendicitis. Awareness of such anomalies can significantly influence intraoperative decision-making and enhance diagnostic accuracy.

## Consent for publication

Written informed consent was obtained from the patient for publication of this case report and accompanying images. A copy of the consent is available upon request.

## Ethical approval

Ethical approval was obtained for the publication of this case report.

## Guarantor

Dr. Suleiman Ayalew Belay

## Research registration number

N/A

## Funding

No funding was received for this case report.

## Author contribution

**Suleiman Ayalew Belay**: Writing - original draft, Data curation, Conceptualization, Resources. **Michael A. Negussie**: Writing - review and editing, Conceptualization.

**Melaku Tessema Kassie**: Writing - original draft, Resources.

**Yishak Abdulsemed**: Data curation, Conceptualization

**Filimon Getaneh Assefa**: Writing - review and editing, Conceptualization

**Fuad Seid Ebrahim**: Data curation, Conceptualization

## Conflict of interest statement

The authors declare no competing financial interests or personal relationships that could influence this work.
